# Evaluation of keratinized tissue augmentation using amnion/chorion allograft vs. autogenous connective tissue in implant therapy: a retrospective study

**DOI:** 10.1007/s00784-025-06173-z

**Published:** 2025-01-25

**Authors:** Nicola De Angelis, Paolo Pesce, Zethy Hanum Kassim, Catherine Yumang, Domenico Baldi, Maria Menini

**Affiliations:** 1 Department of Surgical Sciences and Integrated Diagnostics, Unit of Implant and Prosthetic Dentistry, University of Genoa, Largo R. Benzi 10 , 16121 Genova, Italy; 2 Dental Faculty- Department of Restorative Dentistry, University of Technology MARA Sungai Buloh, Sungai Buloh, Malaysia; 3 Private Practice, Acqui Terme (AL), Province of Alessandria, Italy

**Keywords:** Dental implants, Peri-implant mucosa, Biological width, Soft tissues allograft

## Abstract

**Objectives:**

Successful implant therapy must also ensure the integration of the soft tissues around the crown/abutment emergence profile. The existing literature does not consistently agree on the necessity of a minimal amount of keratinized tissue (KT), though it appears advantageous for the long-term stability and aesthetics of implants. The purpose of this clinical retrospective study is to compare the effectiveness of amnion/chorion membrane and autogenous subepithelial connective tissue in increasing the keratinized mucosa and maintaining it over a 5-year follow-up.

**Methods:**

Twenty patients who had previously undergone implant surgery were included in the study. Ten patients had received the allograft (Group 1) and ten had received autogenous connective tissue (Group 2).An independent examiner retrospectively analyzed the patient records at 7, 15, and 60 days, and five years post-procedure. Data from these observations were collected and analyzed using SPSS Statistics, version 25. Descriptive statistical analysis was conducted.

**Results:**

All patients exhibited an increase in KT. For Group 1, the mean KT width measurements were 1.27 ± 0.46 mm at the initial evaluation, increasing to 2.00 ± 0.38 mm, 2.80 ± 0.78 mm, 3.27 ± 0.80 mm, and 3.01 ± 0.68 mm at 7, 15, and 60 days post-surgery (with prosthesis delivery on day 60), and five years after prosthetic rehabilitation, respectively.

**Conclusions:**

Within the limitations of this retrospective clinical study, both amnion/chorion and connective tissue show significant potential for KT expansion when used in conjunction with implant surgery.

**Clinical relevance:**

The use of allografts, due their low morbidity, and acceptable results should be considered as a viable option for soft tissues augmentations.

## Introduction

The success of dental implants depends on osseointegration and the integration of surrounding soft tissues. These tissues, including keratinized tissue (KT) and alveolar mucosa, are affected by surgical procedures, bone resorption [1], and prosthetic management, which also impact soft tissue morphology and hygiene maintenance [2].

While debates persist, KT around implants is beneficial for aesthetics [3], plaque control [4], and peri-implant tissue health [5]. Clinical studies show that with proper plaque control, the type of peri-implant mucosa has little effect on implant survival [4]. However, a lack of KT can make hygiene maintenance harder [6, 7]. Wider KT is linked to healthier supporting tissues, less bone loss, and reduced inflammation. Insufficient KT increases risks of gingival recession and bone loss, making it essential for stability and aesthetics [8, 9]. 

Comparative studies also indicate that KT is important for reducing plaque accumulation, minimizing mucosal inflammation, and is linked to various immunologic factors [10, 11]. Conversely, insufficient KT around implants increases the risk of gingival recession and crestal bone loss, making adequate KT advantageous for both esthetic and stability purposes [12].

The timing of soft tissue augmentation varies based on clinical needs. It can occur before or during implant placement, during second-stage surgery, or after implant loading. Early interventions often yield better outcomes, as post-loading procedures can lead to complications like mucositis or peri-implantitis [13–15]. 

Recent reviews highlight techniques for improving peri-implant soft tissue. These include KT width expansion using apically repositioned flaps (APPTF) or vestibuloplasty combined with free gingival grafts (FGG) or graft materials like xenogenic (XCM) and allogenic (AMDA) tissues. Volume enhancement methods include autogenous subepithelial connective tissue grafts (SCTG) or soft tissue replacement grafts. APPTF with FGG stands out but requires more surgical time. SCTG and AMDA also effectively increase tissue volume [15]. 

This study evaluates the stability of peri-implant soft tissues five years after augmentation with dehydrated amnion/chorion membranes and autogenous connective tissue grafts.

## Materials and methods

This retrospective study was conducted in full compliance with the Declaration of Helsinki and was approved by the University of Genoa Ethical Committee CERA (nr. 2024/64). Informed consent was obtained from all patients. Additional signed releases were collected for the use of patient images in the study.

A total of 20 records of 20 patients, aged between 28 and 77 years and of mixed gender (12 males and 8 females), were evaluated in the study. The patients included in this analysis had no documented history of chronic periodontitis and were treated at a private practice in Acqui Terme, Italy, in 2018.

The following criteria were used to select records for the retrospective analysis:

- Non-smokers.
- Presence of at least 2 mm of keratinized tissue.

The study included records of cases with at least 2 mm of keratinized tissue documented under one of the following conditions: at the time of implant placement, during second-stage surgery, or at any implant site where mucosal recession with a thin biotype was observed. To provide further clarity, cases were categorized based on the timing of the surgical procedure relative to implant placement. Surgeries performed prior to implant placement aimed to optimize soft tissue conditions before the procedure. Procedures conducted simultaneously with implant placement addressed mucosal or biotype deficiencies during the implant surgery. Surgeries performed after implant placement were carried out either during the healing phase (e.g., at second-stage surgery) or post-loading to manage soft tissue complications or esthetic concerns.

Exclusion criteria included:

- Poor oral hygiene or non-compliance with treatment.
- Presence of systemic diseases, compromised medical conditions, or immunosuppressive conditions contraindicating elective surgery or affecting healing.
- Use of antihypertensive, antilipemic, antiarrhythmic, or anticoagulant medications.
- Pregnancy or lactation.
- Inflammation at implant sites.

**Fig. 1:**
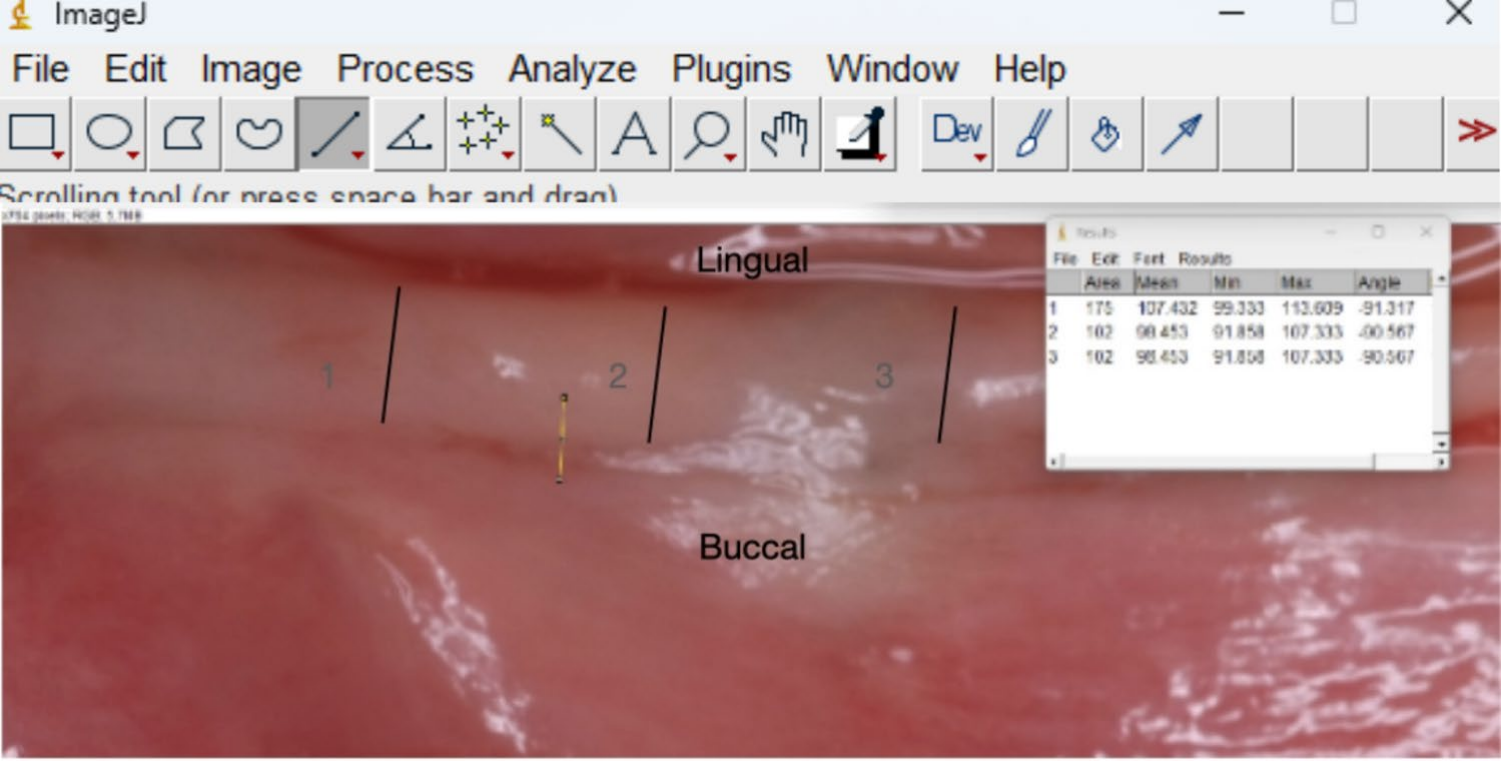
Intraoral photograph after the equalization and measured with ImageJ^®^ software (Rasband, W.S., ImageJ, U. S. National Institutes of Health, Bethesda, MD, USA). Repeated measurements ( distance from buccal to lingual/palatal margin) were retrieved to obtain the mean of the initial dimension of keratinized tissues

Keratinized tissue width measurements were obtained using standardized digital image analysis softwares (CorelDRAW Graphics Suite 2023^®^ and ImageJ^®^). An independent examiner, blinded to the treatment type, performed all the measurements (Fig. 1).

**Fig. 2:**
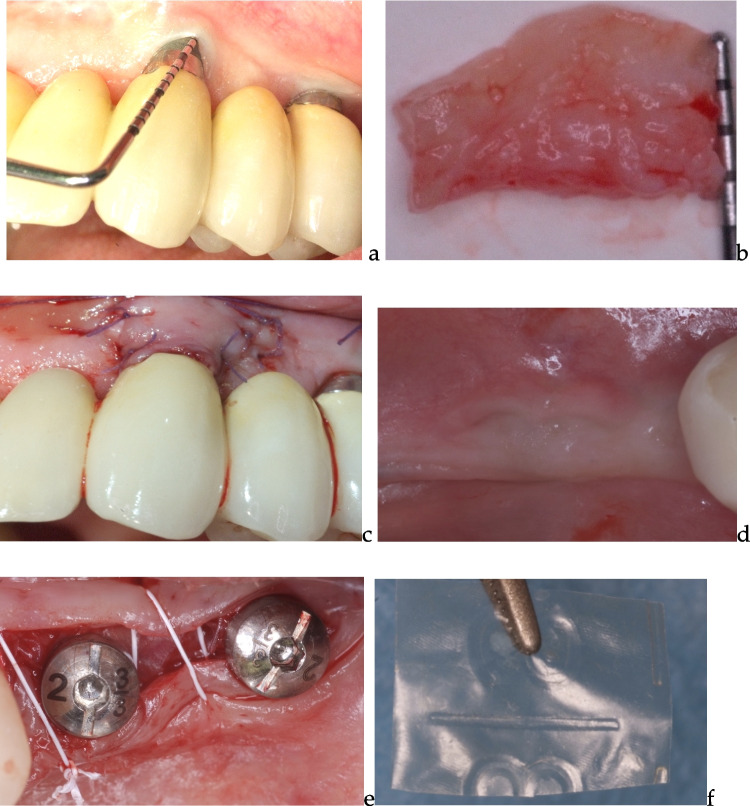
**a** case of previously restored implant with absence of keratinized tissues width and height without signs of inflammation. b autogenous subepithelial connective tissue graft retrieved from the palate and de-epithelized before the insertion (**c**) connective tissue was inserted into an “envelope flap” around the implant (**d**) second stage implant surgery in absence of KT dimension (**e**) healing abutments connected to the implant with the interposition of the amnion-chorion allograft (**f**) amnion-chorion allograft

All patients received standardized oral hygiene instructions and underwent supragingival dental scaling one week prior to surgery. The same specialist (NDA) performed all treatments using 4.3 × 400 surgical head-worn loupes (KS, Carl Zeiss Vision, Jena, Germany). Patients were divided into two groups: Group 1 (*n* = 10) received an allograft, and Group 2 (*n* = 10) received an autogenous sub-epithelial connective tissue graft. The choice of graft material was determined by the operator based on clinical judgment. (Fig. 2a, b,c ).

**Fig. 3:**
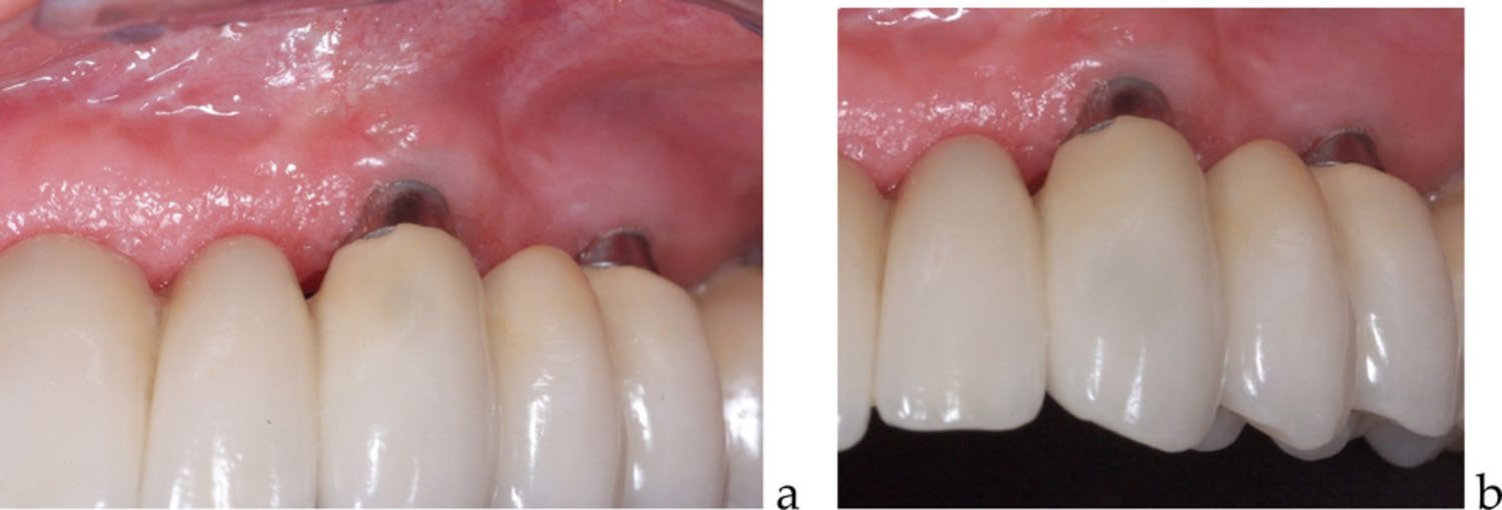
**a** 60 days after the autogenous grafting surgery (**b**) five years control of the same case

**Fig. 4:**
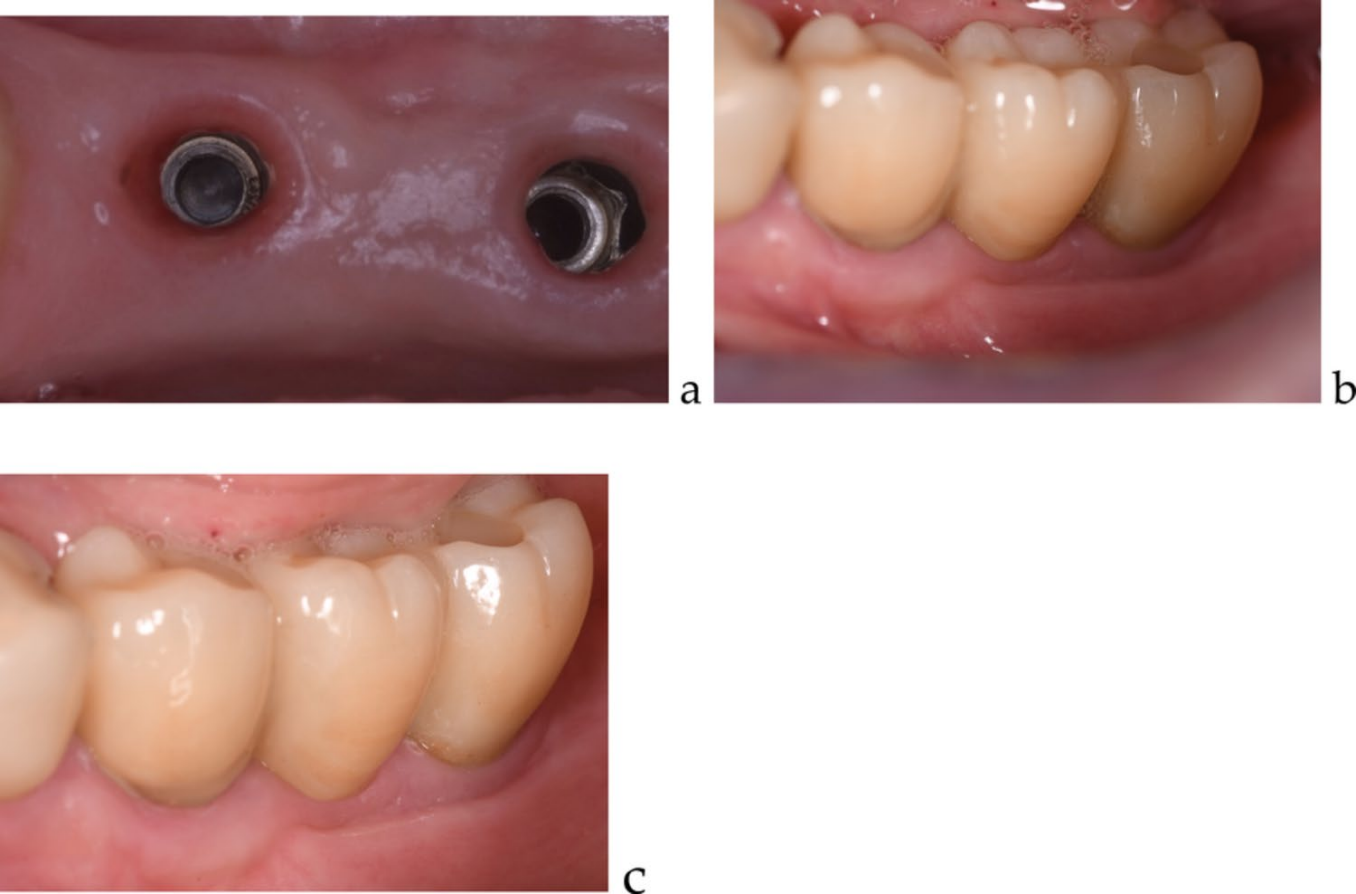
** a** 60 days after the allograft insertion; **b** same case at the prosthetic delivery – 90 days after grafting procedure (**c**) five years control of the same case

Surgical procedures were performed under local anesthesia using Articaine 2% with 1:100000 epinephrine. A full-thickness flap was created, and the graft material (allograft or autogenous) was placed and secured with PTFE sutures. The allograft membrane was left partially exposed during the healing period. Chlorhexidine mouth rinse was recommended post-operatively only for patients treated with autogenous grafts to avoid potential negative interactions with the protein content of the amnion/chorion membrane in the allograft group (Fig. 3a, b) (Fig. 4a, b, c).

Antibiotic prophylaxis was administered as follows [16, 17]:

- Amoxicillin + Clavulanic Acid 875 + 125 mg, 2 g 1 h before surgery, or Azithromycin 500 mg at the same timing.
- Post-operative continuation with Amoxicillin + Clavulanic Acid 1 gram every 6 h or Azithromycin 500 mg for 2 additional days.

Follow-up evaluations were conducted at 7, 15, and 60 days post-surgery.

Participants also received regular dental check-ups and professional oral hygiene treatments at six-month intervals during the follow-up period.

## Statistical analysis

Descriptive statistical analysis was used to get mean and standard deviations for KT width at all time points. In order to analyze the difference in mean KT width at all the different time points, repeated-measurement analysis of variance was performed. For all tests, the level of significance was set at *P* < 0.001 and the confidence interval (CI) was 95%. The software used for the analysis was the IBM SPSS Statistics for iOS, Version 25.0 (IBM Corp., Armonk, N.Y., USA).

## Results

During the 5-year observation period, no implant failures were reported among the study participants. All patients demonstrated an increase in the width of keratinized tissue (KT) over time.

For Group 1 (allograft), the mean KT width measurements were as follows: at baseline, the mean KT width was 1.27 ± 0.46 mm. At 7 days post-surgery, the mean KT width increased to 2.00 ± 0.38 mm. By 15 days post-surgery, the mean KT width further increased to 2.80 ± 0.78 mm. At 60 days post-surgery, coinciding with the time of prosthesis delivery, the mean KT width was 3.27 ± 0.80 mm. Five years post-prosthetic rehabilitation, the mean KT width was measured at 3.01 ± 0.68 mm. (Table 1)

**Table 1:** Group 1** -** Mean keratinized tissue for each measurement time. SD is standard deviation

Time	Mean (SD), mm
Initial	1.27 (0.46)
7 days postsurgery	2.00 (0.38)
15 days postsurgery	2.80 (0.78)
60 days postsurgery/ prosthetic delivery	3.27 (0.80)
5 years after prosthetic delivery	3.01 (0.68)

Group 2 (autogenous sub-epithelial connective tissue) showed similar trends, with data reported in the corresponding table (Table 2). The gains in KT width for both groups were statistically significant (*P* < 0.001) and remained stable over the 5 years of observation.

**Table 2:** Group 2** - **Mean keratinized tissue for each measurement time. SD is standard deviation

Time	Mean (SD), mm
Initial	1.17 (0.43)
7 days postsurgery	1.87 (0.33)
15 days postsurgery	2.30 (0.44)
60 days postsurgery/ prosthetic delivery	3.02 (0.72)
5 years after prosthetic delivery	2.98 (0.56)

These results indicate a statistically significant increase in KT width from the initial evaluation to all post-surgery time points (*P* < 0.001). The most substantial increase in KT width was observed between 7 days post-surgery and the time of prosthesis delivery at 60 days. This increase in KT width remained stable throughout the 5-year follow-up period. ( Table 3 )

**Table 3:** Group 3** -** Comparison of Keratinized Tissue (KT) at each measurement time. Keratinized tissues width increase is noticeable from the initial first measure when compared to post surgical phases and it remains stable over the whole period of observation. Both groups consisted into 10 implants each

Comparison of KT width (mm)	Group 1(10 implants) Mean difference (95% C.I.)	*P*-value	Group 2 (10 implants) Mean difference (95% C.I.)
Initial to 7 days postsurgery	+ 0.733 (–1.096, − 0.371)	< 0.001	+ 0.683 (–1.089, − 0.278)
Initial to 15 days postsurgery	+ 1.533 (–2.194, − 0.873)	< 0.001	+ 1.415 (–1.989, − 0.841)
Initial to prosthetic delivery	+ 2.000 (–2.599, − 1.401)	< 0.001	+ 1.923(–2.456, − 1.301)
7 days postsurgery to 15 days postsurgery	+ 0.800 (–1.414, − 0.186)	0.008	+ 0.803 (–1.423, − 0.184)
7 days postsurgery to prosthetic delivery	+ 1.267 (–1.900, − 0.634)	< 0.001	+ 1.279 (–1.892, − 0.667)
15 days postsurgery to prosthetic delivery	+ 0.467 (–0.876, − 0.057)	0.021	+ 0.433 (–0.799, − 0.067)
Initial to 5 years after prosthetic delivery	+ 1.836 (–2.326, − 1.347)	< 0.001	+ 1.811 (–2.245, − 1.378)

## Discussion

There is a trend shift in implantology from a functional focus on improving function to a greater emphasis on the aesthetics of the prosthetic part [2]. 

This need cannot be addressed solely by prosthetic-driven implant placement; clinicians must consider both biological and aesthetic corrections of the soft tissue to provide a lifelike final prosthetic replacement.

Mucogingival periodontal surgery can improve patient comfort during oral hygiene procedures and lead to better plaque control and less development of mucositis or peri-implantitis. A recent systematic review [15] concluded that implants with soft tissue augmentation showed a high survival rate and a relatively low incidence of peri-implantitis in the medium and long term. Similar to peri-implant tissue health, prosthetic aesthetics would also be enhanced by preventing the abutment or prosthetic margin from showing through the soft tissue. Despite the clinical success of autologous soft tissue [18], its use would increase morbidity due to the need for a second surgical site and increase patient discomfort and acceptance.

The present investigation’s results indicate a similar behavior between autogenous connective tissue and the allograft over five years of observation. A systematic review published in 2021 [19] compared different approaches for peri-implant soft tissue augmentation, including studies using allografts and autogenous connective tissue. The review concluded that a bilaminar approach involving connective tissue or allografts achieved the highest mucosal thickness (MT) gain, while apically repositioned flaps (APF) combined with free gingival grafts (FGG) were most effective for increasing keratinized mucosa width (KMW). Our investigation’s results align with these conclusions, although no significant difference was observed in the progressive reduction of soft tissue width. As described in the methods, only the bilaminar technique was the study’s focus.

The allograft used in this study is a placental-derived amnion/chorion allograft membrane. Amnion/chorion tissue contains various growth factors, including epidermal growth factor, basic fibroblast growth factor, keratinocyte growth factor, transforming growth factor alpha, nerve growth factor, and hepatocyte growth factor, which may promote wound healing and tissue regeneration [20]. The amnion/chorion membrane has been utilized for socket preservation, guided tissue regeneration for periodontal and peri-implantitis defects, maxillary sinus augmentation, guided bone regeneration, and treatment of gingival recession [21, 22], with high success rates in enhancing bone formation and tissue regeneration.

The mean keratinized tissue (KT) gained at the time of prosthetic delivery was 3.27 mm (*P* < 0.01) for allografts and 3.02 mm (*P* < 0.01) for connective tissue, remaining stable at 3.01 mm and 2.98 mm over five years. These results suggest that the presence of numerous growth factors in the allograft may contribute to the rapid growth and long-term maturation of peri-implant tissues.

It is also important to note that the width augmentation achieved immediately after surgery in both groups remained stable over five years, with mean differences of −1.836 mm and − 1.811 mm (*P* < 0.01) for allografts and connective tissue, respectively. This finding does not entirely align with existing evidence, except for a systematic review that focused solely on the stability of autogenous connective tissue grafts [23]. Patient interviews revealed good postoperative satisfaction, and clinical observations showed a good match of the surrounding implant tissue. A recent study by Prakasam et al. [24] compared amnion/chorion membranes with dense polytetrafluoroethylene membranes left intentionally exposed during ridge preservation procedures. They found that patients reported significantly lower postoperative VAS pain scores at amnion/chorion membrane-treated sites, potentially resulting in better quality bone for implant placement.

The preliminary findings published in a 2019 article [25] were corroborated by this retrospective study, suggesting that, despite the small number of included cases, allografts may represent a viable option for peri-implant soft tissue augmentations, demonstrating promising outcomes for enhancing keratinized tissue around implants.

## Conclusions

Despite the limitations of this retrospective study, the procedures investigated show potential for expanding keratinized tissue (KT) when combined with implant surgery or after implant placement. Given the focus on minimally invasive approaches, future research could benefit from exploring the use of allografts or xenografts. Although the small sample size limits the findings, the results suggest promising applications. The increase in KT width observed in this study is consistent when comparing initial measurements to those taken three years later. Additionally, the quality of the tissue, in terms of texture and color, demonstrates stable and complete maturation.

## Data Availability

The data that support the findings of this study are not openly available due to reasons of sensitivity and are available from the corresponding author upon reasonable request. Data are located in controlled access data storage at Genoa University.
